# Dynamic Facial Expressions Prime the Processing of Emotional Prosody

**DOI:** 10.3389/fnhum.2018.00244

**Published:** 2018-06-12

**Authors:** Patricia Garrido-Vásquez, Marc D. Pell, Silke Paulmann, Sonja A. Kotz

**Affiliations:** ^1^Department of Experimental Psychology and Cognitive Science, Justus Liebig University Giessen, Giessen, Germany; ^2^Department of Neuropsychology, Max Planck Institute for Human Cognitive and Brain Sciences, Leipzig, Germany; ^3^School of Communication Sciences and Disorders, McGill University, Montreal, QC, Canada; ^4^Department of Psychology, University of Essex, Colchester, United Kingdom; ^5^Department of Neuropsychology and Psychopharmacology, University of Maastricht, Maastricht, Netherlands

**Keywords:** emotion, priming, event-related potentials, cross-modal prediction, dynamic faces, prosody, audiovisual, parahippocampal gyrus

## Abstract

Evidence suggests that emotion is represented supramodally in the human brain. Emotional facial expressions, which often precede vocally expressed emotion in real life, can modulate event-related potentials (N100 and P200) during emotional prosody processing. To investigate these cross-modal emotional interactions, two lines of research have been put forward: cross-modal integration and cross-modal priming. In cross-modal integration studies, visual and auditory channels are temporally aligned, while in priming studies they are presented consecutively. Here we used cross-modal emotional priming to study the interaction of dynamic visual and auditory emotional information. Specifically, we presented dynamic facial expressions (angry, happy, neutral) as primes and emotionally-intoned pseudo-speech sentences (angry, happy) as targets. We were interested in how prime-target congruency would affect early auditory event-related potentials, i.e., N100 and P200, in order to shed more light on how dynamic facial information is used in cross-modal emotional prediction. Results showed enhanced N100 amplitudes for incongruently primed compared to congruently and neutrally primed emotional prosody, while the latter two conditions did not significantly differ. However, N100 peak latency was significantly delayed in the neutral condition compared to the other two conditions. Source reconstruction revealed that the right parahippocampal gyrus was activated in incongruent compared to congruent trials in the N100 time window. No significant ERP effects were observed in the P200 range. Our results indicate that dynamic facial expressions influence vocal emotion processing at an early point in time, and that an emotional mismatch between a facial expression and its ensuing vocal emotional signal induces additional processing costs in the brain, potentially because the cross-modal emotional prediction mechanism is violated in case of emotional prime-target incongruency.

## 1. Introduction

Emotion is conveyed through different communication channels: facial expressions, tone of voice (emotional prosody), gestures, and others. Moreover, emotional communication in everyday life is dynamic, and we need to constantly monitor the emotional expressions of the people we interact with. However, the majority of past research on emotion perception has focused on single communication channels (e.g., emotional face processing) and on static stimuli rather than dynamic ones—possibly because these stimuli allow for controlled laboratory testing. Recent research has started to tackle the challenges related to multisensory, dynamic emotion processing. The present study follows this important movement and aims to contribute to the field by investigating cross-modal emotional priming with dynamic stimuli.

### 1.1. Cross-modal modulation of emotion processing

In cross-modal emotion perception, at least two processes are involved: cross-modal prediction and audiovisual integration (Jessen and Kotz, 2015). Cross-modal prediction is a mechanism by which information from one modality (e.g., a facial expression) helps predict certain characteristics of the signal in another modality that comes into play later (e.g., a vocal expression). Audiovisual integration refers to the process by which modalities are integrated into a coherent percept. Thus, cross-modal emotional priming, in which visual and auditory information is presented consecutively rather than simultaneously, is a tool to investigate cross-modal prediction independent of audiovisual integration.

Cross-modal emotional priming studies suggest that humans use emotional stimuli from one modality to generate predictions about the other. For example, people are faster and more accurate at deciding whether a facial expression truly reflects an emotion or not when the faces are preceded by emotionally congruent rather than incongruent prosody (Pell, 2005; Pell et al., 2011). These congruency effects also show in event-related potentials (ERPs), in which prime-target congruency modulates an N400-like negativity (Paulmann and Pell, 2010).

In real life audiovisual speech processing, we use a speaker's mouth and face movements to generate predictions about ensuing acoustic stimulation (van Wassenhove et al., 2005; Chandrasekaran et al., 2009). In a similar vein, emotional facial expressions commonly precede vocal emotional input, and may therefore drive a cross-modal emotional prediction mechanism (Jessen and Kotz, 2013; Ho et al., 2015; Kokinous et al., 2015).

Priming research has shown that the presentation of a facial expression affects how subsequent vocal emotional targets are processed: Pourtois and colleagues (Pourtois et al., 2000) used angry or sad facial expressions that were followed by a vocal stimulus with angry intonation. Emotional congruency between facial and vocal expressions affected the amplitude of the auditory N100 component. In a mismatch negativity (MMN) study with the same stimuli, incongruent deviants among congruent standards (or vice versa) triggered an enhanced auditory MMN at 178 ms after sound onset, even though the auditory input was held constant, and participants were instructed to ignore it (de Gelder et al., 1999). Static fearful or happy faces followed by fearful or happy prosody elicited a posterior P2b component in the ERPs, which occurred earlier when face and voice were emotionally congruent rather than incongruent (Pourtois et al., 2002). Thus, vocal emotion processing is influenced by preceding facial information at an early point in time, namely within the first 250 ms of auditory processing. Due to the different nature of these congruency effects and the lack of ERP studies assessing the contextual influence of dynamic face primes on vocal emotion processing, more priming studies are needed to shed light on these processes.

Apart from cross-modal emotional priming studies, researchers have tested audiovisual integration of emotional information, with temporally aligned visual (facial and/or body expressions) and auditory information. This means that the visual information naturally precedes the auditory information, since mouth or body movements are visible first while the auditory information unfolds over time. Comparisons of audiovisual conditions to a purely auditory condition show that emotional prosody is integrated with its preceding visual input within the first 100 ms of vocal emotion processing (Jessen and Kotz, 2011; Kokinous et al., 2015), reflected in an N100 amplitude suppression for audiovisual compared to auditory-only stimuli. This could be due to the visual input leading to predictions about the to-be-expected auditory input, which facilitates auditory processing, for example in terms of temporal predictability of the auditory signal (Vroomen and Stekelenburg, 2010; Jessen and Kotz, 2013; Schröger et al., 2015).

However, temporal predictability of auditory input, based on preceding visual information, is only one type of predictability which modulates the auditory N100 in cross-modal emotion processing. Several ERP studies have manipulated audiovisual emotional congruency within audiovisual integration paradigms while maintaining temporal predictability. In these studies, emotional congruency between face and voice also differentially affected the amplitude of the N100 response (Ho et al., 2015; Kokinous et al., 2015, 2017; Zinchenko et al., 2015). Thus, visual signals are not only used to predict *when* to expect auditory stimulation, but also *what* to expect.

Since these were integration studies in which the visual and auditory tracks were temporally aligned (albeit with visual information available ahead of auditory input), the emotionally incongruent condition implied incongruency of mouth movements and vocal stimulation, in addition to the emotional mismatch between face and voice: Even though the same sounds were used in emotionally congruent and incongruent conditions (e.g., mouth movement for “ah” paired with “ah” sound), mouth movements differ depending on emotion (e.g., the mouth movement while uttering a neutral “ah” is very different from when uttering an angry “ah”). Thus, apart from an emotional mismatch between the visual and auditory channels, there was also a mismatch in mouth movements, which may at least partly account for the reported congruency effects in the N100.

Two studies (Zinchenko et al., 2015, 2017) show that this could in fact be the case: these authors included a comparison between congruent and incongruent audiovisual sounds (e.g., mouth movement for “ah” combined with “ah” sound vs. “oh” sound), while maintaining emotional face-voice congruency. They observed significant congruency effects already in the N100, which shows that a conflict between mouth movement and sound is sufficient to modulate N100 amplitude. It is therefore necessary to complement previous research with priming studies, in which visual and auditory tracks follow each other rather than being temporally aligned. This will help us isolate the processes due to emotional conflict from those related to other types of conflict between visual and auditory information. Moreover, as outlined above, the priming paradigm allows studying cross-modal emotional prediction independent of multisensory integration.

### 1.2. Cross-modal modulation of the P200

In several studies that reported N100 modulations by emotional face-voice congruency, the effects extended into the P200 ERP component (Pourtois et al., 2000; Ho et al., 2015; Kokinous et al., 2015; Zinchenko et al., 2015). In the study by Ho et al. (2015), emotional congruency effects in the N100 were affected by attentional manipulations, while this was not the case for the P200. Two other integration studies reported that audiovisual congruency affected the N100 as a function of visual context (Kokinous et al., 2015) or target category (Zinchenko et al., 2015), while the P200 was globally modulated by face-voice congruency (Kokinous et al., 2015; Zinchenko et al., 2015). In another audiovisual integration study, emotional congruency effects were observed in the P200 component only, but not in the N100 (Zinchenko et al., 2017). These results show that N100 and P200 may reflect different processes in emotional face-voice interactions. Therefore, the present study will also investigate congruency effects in the P200, in order to test whether these two components can be functionally dissociated in dynamic cross-modal emotional priming.

### 1.3. Cross-modal modulation of brain regions

Previous fMRI studies investigating audiovisual emotion processing have reported several audiovisual convergence areas, most notably the posterior superior temporal sulcus and gyrus (STS/STG) (Kreifelts et al., 2007; Robins et al., 2009; Park et al., 2010; Klasen et al., 2011; Watson et al., 2014; Li et al., 2015) and the thalamus (Kreifelts et al., 2007; Klasen et al., 2011).

Some imaging studies have compared emotionally congruent and incongruent audiovisual stimuli. This allows identifying brain regions important for the integration of emotionally congruent signals (Klasen et al., 2012) and also regions associated with higher processing costs due to audiovisual stimulus incongruency. Incongruent emotional stimuli trigger more widespread activations in the brain than congruent ones, which may reflect more effortful processing in the case of emotional incongruency (Klasen et al., 2011; Müller et al., 2011). For example, the cingulate cortex, an area associated with conflict processing, is more activated by incongruent than congruent stimuli (Klasen et al., 2011; Müller et al., 2011).

Because categorizing incongruent emotional stimuli is much harder than categorizing congruent ones (Collignon et al., 2008; Föcker et al., 2011), these activation differences may reflect task difficulty. Therefore, Watson et al. (2013) morphed visual and auditory emotional stimuli independently on an angry-happy continuum in order to manipulate emotion categorization difficulty. They found that after regressing out the variance due to task difficulty, the incongruency effect remained significant in the right STS/STG (Watson et al., 2013). Thus, enhanced activations for incongruent as compared to congruent stimuli reflect more than just task difficulty—they may point to enhanced processing effort while the brain tries to make sense of two stimuli that do not belong together.

While these imaging studies are particularly informative about which brain regions are implicated in cross-modal emotion processing, they fail to clearly link brain structures to the time course underlying cross-modal emotional interactions. The present study thus utilized ERPs to explore when incongruency effects for dynamic emotional stimuli are first observed and adds the ERP source localization technique to link high temporal resolution with potential brain sources.

### 1.4. The present study

We applied a cross-modal emotional priming paradigm with short video clips of facial expressions as primes and emotional pseudo-speech stimuli as targets. We aimed at testing whether facial expressions elicit early congruency effects in the auditory ERPs. Furthermore, since emotional priming studies using static face primes are inconclusive regarding the time point at which audiovisual congruency effects first emerge in ERPs (de Gelder et al., 1999; Pourtois et al., 2000, 2002), we wanted to shed more light on this issue using dynamic face primes, which are more ecologically valid than static facial expressions. We predicted congruency effects at an early time point in auditory processing, namely in the N100 and P200.

Since fMRI exhibits a very good spatial, but low temporal resolution, we localized the neural sources of ERP differences in the present study to explore underlying neural activity at precise points in time. In line with previous neuroimaging research, we expected that incongruent compared to congruent targets would trigger enhanced activations in right STS/STG region, which is modulated by emotional congruency in audiovisual emotion processing irrespective of task difficulty (Watson et al., 2013).

In audiovisual integration studies, emotional faces and voices of one category were paired with neutral faces and voices to construct the emotionally incongruent experimental condition (Ho et al., 2015; Kokinous et al., 2015, 2017; Zinchenko et al., 2015, 2017). Additionally, two priming studies have used angry or sad facial expressions paired with angry voice targets (de Gelder et al., 1999; Pourtois et al., 2000), and one priming study has paired happy and fearful faces and voices (Pourtois et al., 2002). Thus, there are two types of incongruency: pairings of different emotion categories or pairings of emotional with neutral material. The present study used both in order to compare whether the type of incongruency makes a difference in cross-modal emotion processing. We will refer to the combination of neutral primes with emotional targets as “neutral” condition throughout, while the pairings of emotional primes and targets of opposing valence will be referred to as “incongruent” condition in the present paper.

To sum up, the aims of the present study were as follows: (1) to describe the time course of dynamic cross-modal emotional priming with ERPs, (2) to identify underlying neural sources of significant ERP congruency effects, and (3) to test whether the type of cross-modal emotional incongruency (pairings of emotional with neutral stimuli or pairings of stimuli of opposing emotional valence) makes a difference for (1) and (2).

Since previous findings show that part of the processing differences between congruent and incongruent audiovisual emotional stimuli may be due to task difficulty (Watson et al., 2013), we used a gender decision task, which also ensured that participants did not need to consciously focus on the emotional content of our stimuli (Pourtois et al., 2005; Paulmann et al., 2009).

## 2. Methods

### 2.1. Participants

Thirty-six individuals took part in the present experiment. Sample size was based on previous ERP studies (Paulmann and Kotz, 2008; Ho et al., 2015). Two participants had to be excluded from the final sample, one due to technical problems during the EEG measurement and one because of strong noise on almost all scalp electrodes. The remaining 34 participants (17 female) had a mean age of 24.97 years (*SD* = 2.35). All participants reported normal hearing, normal or corrected-to-normal vision and were right-handed (Oldfield, 1971). They received financial compensation for taking part in the experiment. All participants provided written informed consent prior to participating in the experiment. The study was approved by the local ethics committee at the University of Leipzig, and the procedures followed the Declaration of Helsinki.

### 2.2. Stimulus material

**Figure 1:**
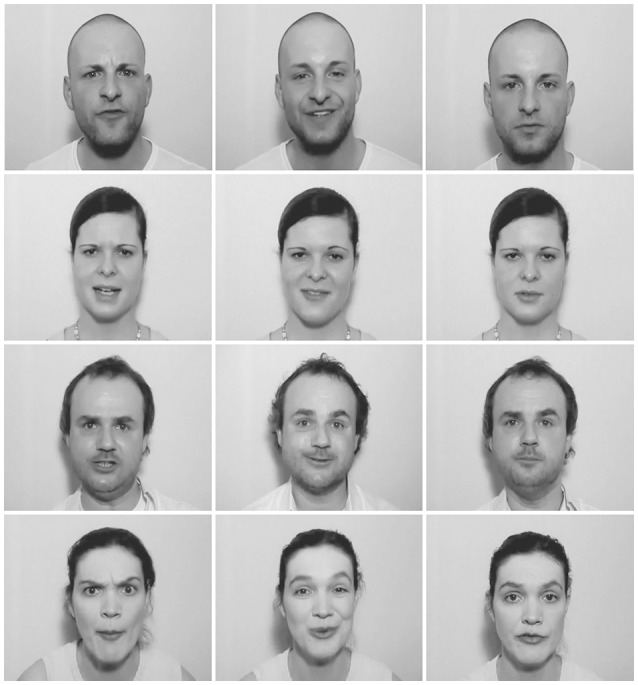
Still example frames from the video primes, displaying angry (left), happy (middle), or neutral (right) facial expressions, one actor per line.

The stimuli consisted of video files without sound, which were used as primes, and audio files, which were used as targets. The videos were black-and-white recordings of four semi-professional actors (two female) showing the face and some surrounding information (hair, neck, etc.; see Figure 1 for examples). Actors were videotaped while uttering happy, angry, and neutral sentences with emotionally matching semantic content and showing the corresponding expressions, which means that mouth movements were visible. To create the stimuli, we removed the audio track from the recordings. Faces were cropped and/or centered when necessary in order to be at the center of the display and to have approximately the same size on screen. Gaze was always directed toward the observer. We cut fragments of 520 ms duration from the middle of the original videos, such that the full facial expression was visible from the first video frame on. The 520 ms prime duration was based on considerations that prime durations or prime-target SOAs below 300 ms may lead to reversed priming effects (Bermeitinger et al., 2008; Paulmann and Pell, 2010), which we wanted to prevent. Moreover, dynamic facial expressions elicit the strongest ERP responses within the first 500 ms of processing (Recio et al., 2014), and we aimed to avoid an overlap of these with early vocal emotion processing. This prime duration is also roughly comparable with the temporal precedence of visual information in cross-modal emotional integration studies (Ho et al., 2015; Kokinous et al., 2015). Due to the 40 ms frame length, video duration can only be a multiple of 40, which is why we chose the seemingly arbitrary video duration of 520 ms.

Video selection for the present experiment was based on results from a validation study with 28 participants who were not recruited for the present study (see Garrido-Vásquez et al., 2016 for details). Based on these data, 240 video stimuli (3 emotional categories × 4 actors × 20 videos) were selected, which in the validation study were recognized on average 2.7 times better than chance (chance level: 33%). We used a rather large number of different stimuli to reflect the natural variability inherent in emotion expressions.

The audio files were happy and angry sentences uttered in pseudo-speech by the same four actors who appeared in the videos. Thus, semantic content could not be derived from the sentences, but they nevertheless matched German phonotactic rules and all had the same syntactic structure (e.g., “Hung set das Raap geleift ind nagebrucht.”). Duration of these stimuli was approximately 3s. The sentences were digitized at a 16-bit/44.1 kHz sampling rate. They were normalized to peak amplitude to ensure an equal maximum volume for all stimuli. Recognition of these materials was also pre-tested on a different sample of 24 participants, and for each actor and category we selected the 30 highest-ranking stimuli, resulting in 240 happy and angry sentence stimuli to be paired with the videos. Average recognition rates were more than five times higher than chance (chance level: 14%). These stimuli have been used in prior research (e.g., Paulmann et al., 2010; Garrido-Vásquez et al., 2013).

### 2.3. Procedure

We conducted the EEG experiment in an electrically-shielded and sound-attenuated room. Participants were sitting at a distance of approximately 100 cm from the computer screen. Videos were presented centrally at an image resolution of 720 × 576 pixels, and the faces subtended a visual angle of approximately three degrees to each side. We used the MPEG-4 codec to optimize timing, and frame rate was 25 frames per second. Auditory stimuli were presented at a constant and comfortable listening level. The experiment was programmed in Presentation (Neurobehavioral Systems, San Francisco, USA).

Each trial started with a black fixation cross on a gray background (1,000 ms), followed by the video prime (520 ms). Immediately after video offset, the fixation cross became visible again and the auditory target played via loudspeakers located left and right to the screen. Identity of the actor in the video and in the audio file always matched within a trial. The fixation cross stayed on screen until the end of the auditory stimulus and was then replaced by a black question mark, prompting participants to indicate whether a female or male speaker had been presented. Answers were provided by means of a button box, and half of the participants pressed the left button for “female” and the right button for “male,” while the other half proceeded vice versa. Participants were instructed to answer as fast and accurately as possible. After the button press, a gray blank screen showed up (2,000 ms), and then the next trial began.

The 240 trials were divided into four blocks of 60 trials each and presented in a pseudo-randomized order that differed for each participant. In one third of the trials, prime and target were emotionally congruent (happy-happy or angry-angry), in another third they were incongruent (happy-angry or angry-happy), and yet another third were neutral trials (neutral-happy or neutral-angry). Our randomization allowed for a maximum of three consecutive trials with the same actor, same prime category (happy, angry, or neutral), or the same prime-target relationship (congruent, incongruent, neutral). Unrelated to the current investigation, within the same experimental session we also tested the reverse prime-target order, i.e., with pseudo-sentences as primes and videos as targets (results not reported here). Half of the participants started with the video-as-prime-condition, and the other half with the audio-as-prime condition. Total run-time of the experiment was approximately 60 min including breaks.

### 2.4. Data acquisition and analysis

We recorded the EEG data from 61 scalp electrodes mounted in an elastic cap according to the extended international 10-10 system. Data were referenced to the average of all electrodes online and re-referenced to the mean activity at left and right mastoids offline. Recording was accomplished with a bandpass between DC and 140 Hz, and the data were digitized at 500 Hz. Four electrodes (two horizontal, two vertical) were applied to register eye movements during the measurement, and the ground electrode was placed on the sternum. Electrode resistance was below 5kΩ.

We used FieldTrip (Oostenveld et al., 2011) running on Matlab (The Mathworks, Natick, USA) to further process the EEG data offline. Continuous data were filtered with a highpass filter at a cutoff frequency of 1 Hz (1,762 points, Blackman window, finite impulse response filter). This filter did not only remove slow drifts, but it also served to replace the baseline, because we were interested in measuring ERPs elicited by the prosodic targets, but obtaining a clean pre-stimulus baseline was not possible due to the prime, which directly preceded the target (see, e.g., Jessen and Kotz, 2011). After cutting the data into epochs of 1,000 ms duration and time-locked to target onset, we first manually inspected all trials for atypical artifacts, which were rejected. Then, the data were subjected to an independent component analysis (ICA) to identify components associated with eye movements or other artifacts (electrocardiographic artifacts or noisy electrodes). These components were removed from the data, and then the ICA-corrected data were inspected manually again in order to reject any trials that still contained artifacts. Furthermore, all trials with incorrect or missing responses were excluded from the data. In total, 21% of all trials were excluded based on these criteria. We applied a 40 Hz lowpass filter on the EEG data for the visual ERP displays.

The clean 1,000 ms epochs were averaged according to target emotion (happy, angry) and congruency with the prime (congruent, incongruent, neutral). Time windows and electrode sites for the N100 and P200 analysis were defined based on the “Collapsed localizers” procedure (Luck and Gaspelin, 2017), which consists of averaging all experimental conditions together and then identifying electrodes and time windows at which the component of interest is maximal. The selected electrodes, at which both N100 and P200 were maximal were: FC3, FCz, FC4, C3, C1, Cz, C2, C4, CP3, CPz, and CP4. Time windows selected according to this procedure were: 80-130 ms post-target onset for the N100 and 180–250 ms post-target onset for the P200. For the ERP amplitude analyses, we averaged the data across the respective time windows and all included electrodes. Furthermore, the selected electrodes and time windows were also used to extract N100 and P200 peak latency for the ERP latency analysis. For both amplitude and latency data, values were submitted to a 3 (congruency) × 2 (emotion) repeated-measures ANOVA. Mauchly's test for sphericity was insignificant for all effects; therefore we used the original degrees of freedom in the ANOVA.

### 2.5. Source reconstruction

In case of significant ERP results, we conducted a source reconstruction on the respective ERP time window to uncover neural generators of the effects. These analyses were realized in SPM12 (http://www.fil.ion.ucl.ac.uk/spm/software/spm12/). Individual electrode locations obtained via digitization were co-registered with SPM's standard template head model in MNI space with a cortical mesh of 8,196 vertices. We constructed the forward model using the Boundary Elements Method implemented in SPM, which is based on realistic head geometry and takes into consideration the different conductor properties of brain tissues. We inverted the data for all conditions and participants together, using the minimum norm estimation algorithm (IID). The results were smoothed with a Gaussian kernel full-width half-maximum (FWHM) of 12 mm.

The six average images (one per condition) for each participant were taken to second-level analyses. We conducted two-sided *t*-tests for paired samples in order to compute contrasts between the congruent, incongruent, and neutral conditions collapsed across angry and happy targets. We also computed these contrasts separately according to target emotion (e.g., angry congruent vs. angry incongruent). All contrasts were calculated in both directions (e.g., congruent > incongruent and incongruent > congruent). Results that survived family-wise error correction at an alpha level of *p* < 0.05 were deemed significant.

## 3. Results

Behavioral data were not further analyzed, since gender decision performance was at ceiling.

### 3.1. N100 and P200 amplitude

**Figure 2:**
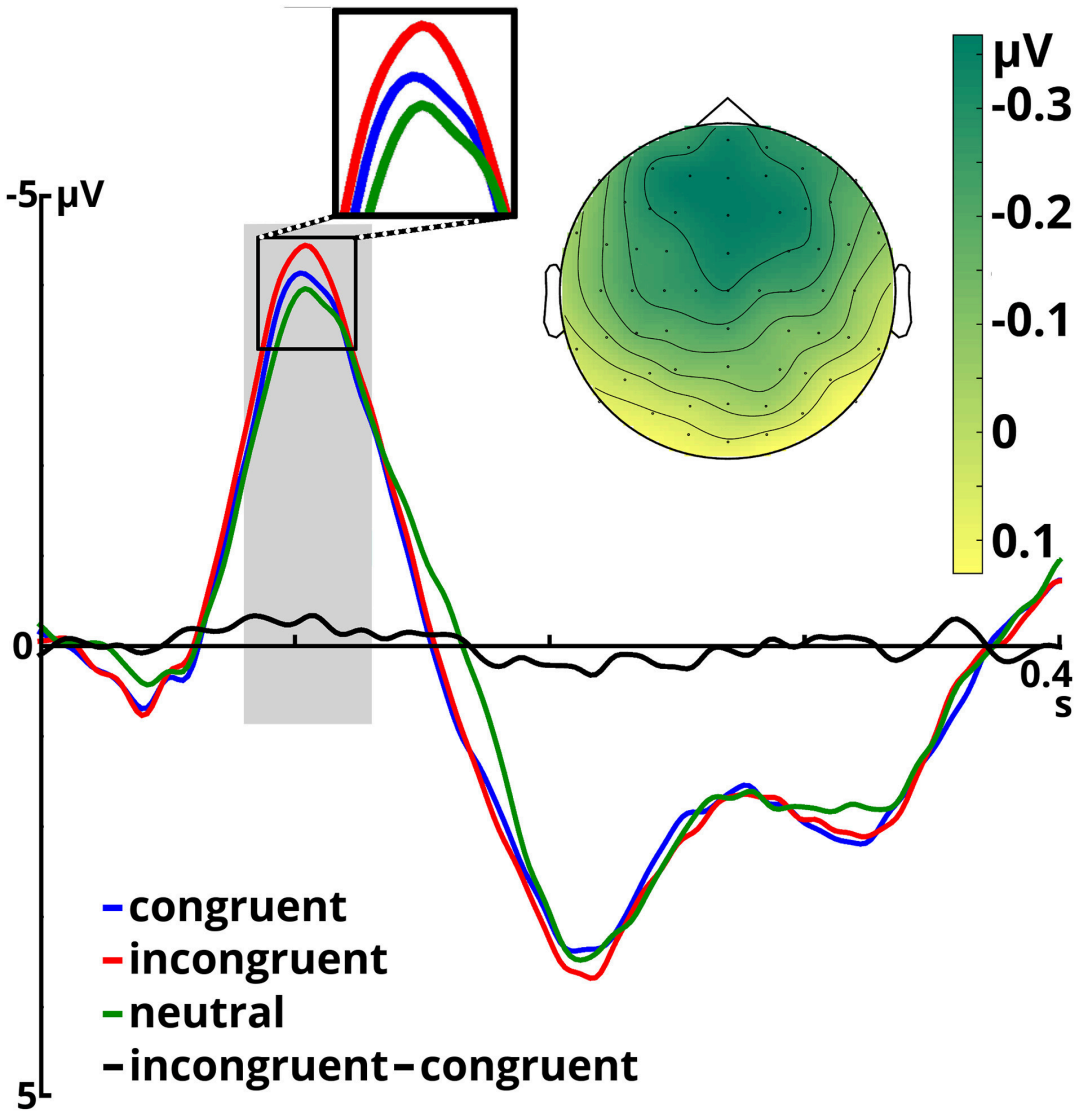
Event-related potentials averaged over all included electrodes for the three congruency conditions and the incongruent - congruent difference, time-locked to target onset. The time window for N100 analysis is shaded in gray. The scalp potential map shows the incongruent - congruent difference in the N100 time window.

Figure 2 shows N100 and P200 time-locked to target onset in the three congruency conditions. An overview of means and standard deviations both for ERP amplitude and latency values in all six conditions is provided in Table 1.

**Table 1:** ERP amplitude and latency results.

**N100 MEAN AMPLITUDE**			
**Intonation**	**Prime**	**Mean voltage (μV)**	**SD**
Angry	Congruent	-3.51	1.67
Angry	Incongruent	-3.94	1.85
Angry	Neutral	-3.53	1.79
Happy	Congruent	-3.29	1.97
Happy	Incongruent	-3.42	1.98
Happy	Neutral	-3.03	2.11
**N100 PEAK LATENCY**			
**Intonation**	**Prime**	**Peak latency (ms)**	**SD**
Angry	Congruent	105	11.38
Angry	Incongruent	102	9.74
Angry	Neutral	107	10.10
Happy	Congruent	104	9.42
Happy	Incongruent	104	11.34
Happy	Neutral	108	11.58

The ANOVA on the N100 time window yielded a significant main effect of congruency, *F*_(2, 66)_ = 4.899, *p* = 0.01, η^2^*p* = 0.129. In the incongruent condition (*M* = −3.68, SD = 1.80) N100 amplitudes were larger than in the congruent condition (*M* = −3.40, *SD* = 1.73), *t*_(34)_ = 2.491, *p* = 0.018. The same held true when comparing the incongruent to the neutral condition (*M* = −3.28, *SD* = 1.85), *t*_(34)_ = 3.046, *p* = 0.005. Amplitudes in the congruent and neutral condition did not significantly differ (*p* = 0.407). Furthermore, we observed a significant main effect of emotion, *F*_(1, 33)_ = 12.108, *p* = 0.001, η^2^*p* = 0.268. Angry prosody (*M* = −3.66, *SD* = 1.66) elicited higher N100 amplitudes than happy prosody (*M* = −3.25, *SD* = 1.88). The interaction between both factors was insignificant (*p* ≥ 0.571).

P200 amplitude was not significantly modulated by congruency or emotion (*p*s > 0.150).

### 3.2. N100 and P200 peak latency

N100 latency was significantly modulated by target congruency, *F*_(2, 66)_ = 8.976, *p* < 0.001, η^2^*p* = 0.214. This component peaked later in the neutral condition (*M* = 108 ms, *SD* = 9.50) than in the congruent (*M* = 105 ms, *SD* = 9.58), *t*_(34)_ = 2.610, *p* = 0.014, and incongruent (*M* = 103 ms, *SD* = 10.06), *t*_(34)_ = 4.246, *p* < 0.001 conditions. The latter two did not significantly differ (*p* = 0.098). The main effect of emotion and the congruency x emotion interaction were not significant (*p*s ≥ 0.299).

We failed to find any significant main effects or interactions for P200 peak latency (*p*s ≥ 0.163).

### 3.3. Source reconstruction

**Figure 3:**
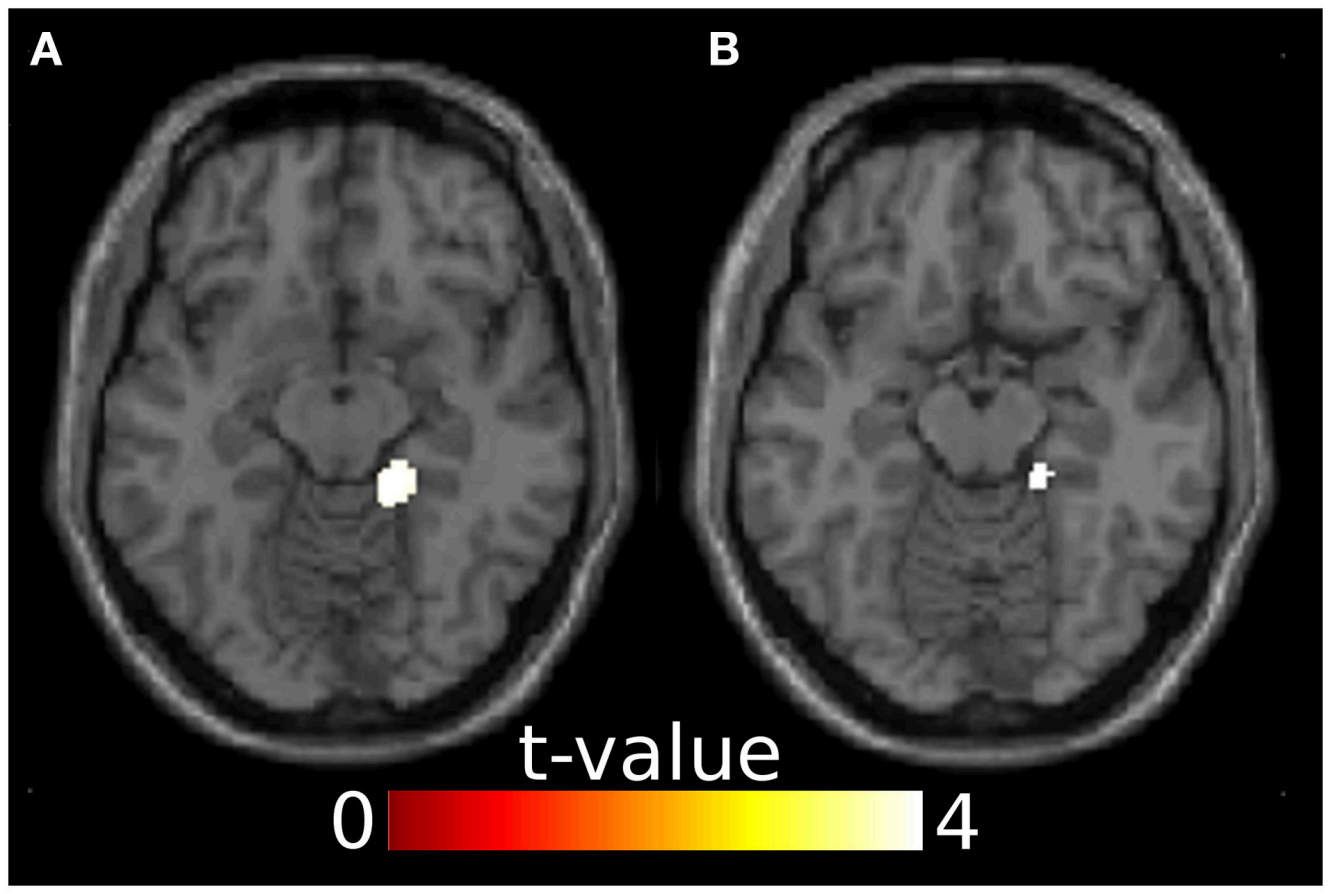
Results from the source reconstruction analysis showing significant clusters in the right parahippocampal gyrus. **(A)** Incongruent > congruent, z = -12. **(B)** Anger incongruent > anger congruent, z = -14. Images are thresholded at *p* < 0.05 family-wise error corrected.

Since ERP analyses revealed significant effects only for the N100, we restricted source reconstruction to this component and to the alpha frequency range (Herrmann et al., 2014). Incongruent targets triggered significantly stronger activations in the right parahippocampal gyrus (PHG) than congruent targets. The right PHG was also more active in angry incongruent compared to angry congruent trials. None of the other contrasts survived the threshold of *p* < 0.05 family-wise error corrected. Congruently primed targets did not elicit additional activations when compared to incongruently primed ones, even at a very lenient threshold of *p* < 0.01 (uncorrected). See Table 2 and Figure 3 for results of the source reconstruction analysis.

**Table 2:** N100 source reconstruction results.

**Contrast**	**MNI peak voxel**	**Z**	**Cluster size**	***p*^a^**	**Region**
ic > c	16 -36 -12	4.04	145	0.025	Right PHG
ang ic > ang c	18 -34 -14	3.90	28	0.041	Right PHG

*c, congruent; ic,incongruent; ang, anger; PHG, parahippocampal gyrus*.

a *Family-wise error corrected (p-value and cluster size)*.

## 4. Discussion

In the present study we investigated cross-modal emotional priming with videos showing happy, angry, or neutral facial expressions followed by happy or angry emotional prosody. We successfully replicated early audiovisual congruency effects in the N100 ERP component. Building on unimodal and multimodal priming studies with static facial expressions (Pourtois et al., 2000; Werheid et al., 2005), we showed that dynamic emotional face primes successfully establish an emotional context under which subsequent emotional targets are evaluated. By including a neutral prime category in addition to the incongruent one, we were able to show that these two types of prime-target incongruency elicit different processes, which we will discuss in more detail below. Moreover, the right PHG was more activated during the processing of incongruently, rather than congruently primed auditory targets within the N100 time window.

### 4.1. N100 enhancement in the incongruent condition

Emotional priming affected auditory processing at an early time point, namely in the N100. This is in line with several previous studies, which have shown such early emotional congruency effects (Pourtois et al., 2000; Werheid et al., 2005; Ho et al., 2015; Kokinous et al., 2015, 2017; Zinchenko et al., 2015), with findings from audiovisually presented congruent and incongruent human speech sounds (Zinchenko et al., 2015, 2017), and with studies comparing unimodal to audiovisual emotion (Jessen and Kotz, 2011; Kokinous et al., 2015) and speech processing (van Wassenhove et al., 2005).

This evidence suggests that information from auditory and visual domains can be combined within the first 100 ms of auditory processing, possibly facilitated through a cross-modal prediction mechanism (van Wassenhove et al., 2005; Jessen and Kotz, 2013; Ho et al., 2015). Commonly, emotional facial expressions temporally precede vocal expressions of emotion in human interactions and thus allow us to predict some characteristics of the ensuing auditory signal, such as its temporal onset and some acoustic properties. However, if for example an angry face precedes a vocal expression of happiness, the prediction is violated, leading to enhanced processing costs. In our study, these were reflected in an enhanced N100 amplitude and right PHG activation, which we will discuss in more detail below.

N100 enhancement in the incongruent condition indicates that emotional significance in the voice could at least partly be extracted already during the first 100 ms of auditory processing. When vocal emotion is presented in isolation (i.e., unimodally), emotional significance is thought to be extracted after approximately 200 ms, in the P200 component (Schirmer and Kotz, 2006; Paulmann and Kotz, 2008; Pell et al., 2015), while earlier steps are associated with sensory processing (Schirmer and Kotz, 2006). However, some studies using unisensory vocal emotional stimuli have also reported emotion effects in the N100 (Liu et al., 2012; Kokinous et al., 2015; Pinheiro et al., 2015), although these may be triggered by low-level features of the stimuli (Schirmer and Kotz, 2006). In the current study low-level features are an insufficient explanation for the N100 modulations, because congruency effects did not differ as a function of target emotion and were modulated only by the prime-target relation *per se*. We could therefore hypothesize that emotional information in the face (e.g., a smile) leads to the prediction that the ensuing vocal stimulus will be of a certain quality (e.g., rather high-pitched) and thereby facilitates auditory processing if this prediction is fulfilled.

### 4.2. Absence of significant congruency effects in the P200

In contrast to audiovisual emotion studies that reported emotional congruency effects also in the P200 (Pourtois et al., 2000; Ho et al., 2015; Kokinous et al., 2015; Zinchenko et al., 2015) or exclusively in the P200 (Balconi and Carrera, 2011; Yeh et al., 2016; Zinchenko et al., 2017), we failed to observe any significant ERP differences for this component. We argue that in studies showing congruency effects only in the P200, different mechanisms may have shifted emotional congruency effects toward the P200: Balconi and Carrera (2011) used static facial displays whose onset was temporally aligned to their prosodic stimuli; therefore participants may have needed longer than in other studies for combining auditory and visual cues. This is supported by a study by Paulmann et al. (2009), who used static facial expressions whose onset was aligned to (congruent) emotional prosody onset. They found a P200 amplitude reduction for audiovisual compared to unimodal stimulation, but no N100 effects. Yeh et al. (2016) used bodily expressions, which may be a less reliable predictor of vocal emotional expressions than a face (although these authors did show N100 suppression during audiovisual compared to auditory processing, but irrespective of congruency). Furthermore, identity mismatches between the visual and auditory tracks could have played a role in their study, because the materials came from different stimulus databases. Zinchenko et al. (2017) employed happy and neutral stimuli, and probably the conflict between happy and neutral cues is not big enough to trigger any congruency effects in the N100, but shifts them to the P200. This partly aligns with our study, in which we failed to show N100 differences between congruently and neutrally primed prosodic stimuli. Thus, methodological differences between studies may lead to a temporal shift of cross-modal interactions because participants take longer to process cross-modal emotional congruency.

In the present study, congruency effects started to emerge early, but were rather short-lived. We propose that the lack of P200 effects may follow from the gender decision task we used: As the face was always presented first and identity of the actor in the video and in the audio always matched within a trial, it was sufficient to make the gender decision based on the face only. Even though we did not instruct participants to do this, they may have realized the identity match after a few trials. Thus, it is likely that they rather attended to the face than to the voice in the present experiment, which is supported by the fact that people often prefer emotional information from faces over information from voices or that facial expressions are more difficult to ignore than vocal expressions (Collignon et al., 2008; Klasen et al., 2011; Ho et al., 2015). Moreover, the task we used did not draw attention to the emotional quality of the stimuli. Studies that show both N100 and P200 modulations by cross-modal emotional congruency (Ho et al., 2015; Kokinous et al., 2015; Zinchenko et al., 2015) have at least in part used tasks that draw attention to the emotionality of the voice. It is, however, unclear why congruency effects extended into the P200 in the study by Pourtois et al. (2000), who instructed participants to attend to the faces and ignore the voices, or why cross-modal emotional congruency affected the MMN in the study by de Gelder et al. (1999)—one explanation could be that they used static facial expressions, while the dynamic primes in our study were processed more quickly and efficiently (Mayes et al., 2009), leading only to short-lived congruency effects in the auditory ERPs. Future research manipulating cross-modal emotional congruency should experiment with different task instructions and dynamic vs. static stimuli to shed more light on this issue. In any case, our results are in line with other studies that suggest that N100 and P200 can be functionally dissociated during cross-modal emotion processing (e.g., Ho et al., 2015; Kokinous et al., 2015).

### 4.3. Role of the right parahippocampal gyrus in cross-modal emotional priming

Source localization revealed that in incongruent compared to congruent trials, the right PHG was engaged in the N100 time window. This difference was apparently driven by angry target stimuli, because the angry incongruent > angry congruent contrast was significant in the right PHG while the happy incongruent > happy congruent contrast was not.

Two studies comparing bimodal emotional face-voice combinations to unimodal conditions have reported enhanced right PHG activation (Park et al., 2010; Li et al., 2015). PHG is also more active when affective pictures are combined with emotional music as compared to when the pictures are presented in isolation (Baumgartner et al., 2006). These three studies (Baumgartner et al., 2006; Park et al., 2010; Li et al., 2015) used only congruent audiovisual inputs. Thus, the right PHG may be involved in binding emotional information from different modalities, and its enhanced activation for incongruent targets in the N100 window may reflect its stronger recruitment when facial and vocal information mismatch.

There is not much evidence on how the PHG relates to early auditory processing, but it has been associated with auditory deviance detection in oddball tasks during the N100 (Mucci et al., 2007; Karakaş et al., 2009). This evidence is in line with our findings: In the oddball task, in which a sequence of frequent standard stimuli is sometimes interrupted by deviant stimuli, participants will generally expect the standard tone because it occurs with greater likelihood than the deviant. Thus, in case of a deviant the prediction is violated similarly to the emotional prediction in incongruent trials in our experiment. This leads to enhanced processing effort, which engages the PHG. According to a relatively recent account (Aminoff et al., 2013), the PHG codes for contextual associations, and in the context of emotion it facilitates emotion understanding and expectations, which perfectly fits with the current results—if face and voice are emotionally incongruent, then the face is a non-reliable contextual cue, leading to more effortful processing of the voice target in the right PHG.

Interestingly, fMRI studies comparing incongruent to congruent audiovisual emotion processing have not reported right PHG activation (Klasen et al., 2011; Müller et al., 2011). We propose that this could be due to the early nature of these activations, which are potentially hard to capture with fMRI. On the other hand, our source localization results converge with those from fMRI studies in that there are no regions found to be more active in the congruent compared to the incongruent condition (Klasen et al., 2011; Müller et al., 2011).

The question arises as to why source localization failed to reveal any activation foci in the STS/STG, while this region has been reported in the neuroimaging literature comparing incongruent to congruent audiovisual emotion stimuli (Kreifelts et al., 2007; Robins et al., 2009; Park et al., 2010; Klasen et al., 2011; Watson et al., 2013, 2014). One potential reason may be time course. Due to low temporal resolution in fMRI, we do not know when this region comes into play. According to a network analysis of audiovisual emotion processing (Jansma et al., 2014), PHG may modulate STS/STG activity unidirectionally; therefore the distinctive activation patterns based on prime-target congruency in STS/STG may come into play later in time.

### 4.4. The neutral prime condition: just another incongruent condition?

In the present study we used both an incongruent condition, with happy primes and angry targets or vice versa, and a neutral condition, in which a neutral facial expression was followed by happy or angry prosody. This allowed testing whether emotional information preceded by neutral information leads to the perception of audiovisual incongruency comparable to the incongruent condition.

N100 amplitude was significantly enhanced in the incongruent compared to the neutral and congruent conditions, which did not significantly differ from each other. Thus, prime-target incongruency may have triggered additional processing effort in the brain, while this was not the case in the neutral prime condition. Moreover, the incongruent condition triggered stronger right PHG activation than the congruent one, which supports the higher processing effort interpretation. Even though this did not apply for the incongruent > neutral contrast, a more liberal threshold of *p* < 0.001 (uncorrected) would yield right PHG activation in this comparison. Thus, we can cautiously state that processing effort in the incongruent condition was also higher than in the neutral condition.

In contrast to our ERP results for the neutral priming condition, other studies reported congruency effects in the auditory N100 when a neutral face preceded angry prosody (Ho et al., 2015; Kokinous et al., 2015, 2017). One potential explanation for this effect could be the task. Kokinous et al. (2015) used an emotion-related task; their participants were asked to indicate whether the prosodic stimulus expressed anger or not. Ho et al. (2015) employed four different tasks: participants judged (1) emotionality in the voice, (2) emotionality in the face, (3) emotional face-voice congruence, or (4) temporal synchrony between face and voice channels. All but the last task were thus emotion-related, and in all but the last task did the authors report that the N100 in response to angry voices was modulated by the fact whether the face was angry or neutral. Thus, the results from the synchrony judgment task Ho et al. (2015) converge with our findings, which we gathered using a gender decision task, a task unrelated to emotion. Attention to the emotional quality of a stimulus may therefore be necessary in order for neutral face primes to trigger congruency effects in the N100. However, we found longer N100 peak latencies for the neutral prime condition compared to the congruent and incongruent conditions. This could mean that an emotional prime speeds up target processing, regardless of congruency, which is in line with previous findings (e.g., Burton et al., 2005).

Generally speaking, neutral stimuli may be less informative for cross-modal prediction because they are not as clear as emotional expressions (Jessen and Kotz, 2015). This is in accord with the results from our pre-test, in which a different set of participants watched and categorized the videos used here. While we obtained very high hit rates for the angry and happy videos (98 and 94%, respectively), neutral videos were recognized with 78% accuracy only. These data support the notion that neutral stimuli are more ambiguous than emotional ones.

The rather small differences between the neutral and congruent conditions in the present study may also be due to our design: If we consider the neutral priming condition an incongruent condition, as has been the case in previous research (e.g., Ho et al., 2015; Kokinous et al., 2015, 2017; Zinchenko et al., 2015, 2017), then two thirds of all trials were incongruent. Due to this imbalance, the neutral trials potentially triggered less conflict than when prime and target were of opposing valence, and the rather small differences between the neutral and congruent conditions in the present study could be attributed to this fact. Moreover, the neutral prime was never paired with a neutral target in the current study and was therefore not suitable to predict ensuing acoustic stimulation. However, it is currently unclear whether prime-target assignments within an experiment can induce transient changes in cross-modal prediction during emotion processing and override long-term associations (Jessen and Kotz, 2013). If these transient changes exist, then the proportion of incongruent among congruent trials in an experiment should influence congruency effects, an issue that still needs to be investigated.

### 4.5. Limitations

As outlined in the previous paragraph, it is not clear whether the presence of the neutral condition in addition to the congruent and incongruent ones may have affected the current results. Moreover, we tested only two emotional categories (happy and angry), which are furthermore of opposing valence (positive and negative). Thus, we cannot say whether our findings are attributable to the emotions *per se*, or to valence effects. It is also not clear why significant effects in the P200 were absent, and whether quicker and more efficient processing of the dynamic prime stimuli or task effects are a suitable explanation for this observation. These limitations to our study will have to be addressed by future research.

## Conclusion

The present study employed a cross-modal emotional priming paradigm with dynamic facial expressions. We showed that priming with a dynamic emotional facial expression affects vocal emotion processing already in the N100 ERP component. An enhanced N100 component as well increased right PHG activation to incongruent targets indicate that processing incongruently primed vocal emotional targets was more effortful than when they had been congruently primed, which may be due to the violation of cross-modal predictions. Our data are in line with many ERP studies showing that audiovisual emotional information is already combined within the N100 time window.

## Author contributions

PG-V, MP, SP, and SK designed research. PG-V conducted the experiment and analyzed the data. PG-V, MP, SP, and SK wrote the paper.

### Conflict of interest statement

The authors declare that the research was conducted in the absence of any commercial or financial relationships that could be construed as a potential conflict of interest.
